# Greater than the sum of the parts: a qualitative content analysis of what constitutes a good treatment in the inpatient setting

**DOI:** 10.1186/s12913-022-07834-5

**Published:** 2022-04-27

**Authors:** Cosima Locher, Sarah Buergler, Nadja Heimgartner, Helen Koechlin, Heike Gerger, Jens Gaab, Stefan Büchi

**Affiliations:** 1 Clinic for Psychotherapy and Psychosomatics Hohenegg, Meilen, Switzerland; 2 Department of Consultation-Liaison Psychiatry and Psychosomatic Medicine, University Hospital Zurich, University of Zurich, Zurich, Switzerland; 3 Faculty of Health, University of Plymouth, Plymouth, UK; 4 Division of Clinical Psychology and Psychotherapy, Faculty of Psychology, University of Basel, Basel, Switzerland; 5 Division of Child and Adolescent Health Psychology, Department of Psychology, University of Zurich, Zurich, Switzerland; 6 Department of Psychosomatics and Psychiatry, University Children’s Hospital Zurich, Zurich, Switzerland; 7 Department of Anesthesiology, Critical Care and Pain Medicine, Boston Children’s Hospital, Harvard Medical School, Boston, USA; 8 Department of General Practice, Erasmus MC University Medical Center, Rotterdam, The Netherlands

**Keywords:** Good treatment, Qualitative content analysis, Inpatient setting, Psychotherapy

## Abstract

**Background:**

The evaluation of psychotherapy is guided by established concepts, such as efficacy and effectiveness, and acceptability. Although these concepts serve as valid proxies, little is known about corresponding criteria for those directly involved in this treatment. This study aimed to explore inpatients’ and health professionals’ definitions of a good treatment in the inpatient setting.

**Methods:**

Fifteen semi-structured interviews were conducted in a private psychiatric clinic in Switzerland and structured by qualitative content analysis. Different subsamples of the inpatient setting (patients *N* = 5; psychiatrists *N* = 5; other health professionals *N* = 5) were interviewed.

**Results:**

In total, 546 text passages were grouped in 10 superordinate categories and identified as relevant for the concept of a good treatment. Participants stressed patient-specific (i.e., new insights; basic attitudes), treatment-specific (i.e., therapy methods and expertise; treatment success; therapy setting), and relationship-based (i.e., communication and feedback; relationships within the clinical setting; overcoming challenges and hurdles) components that are indispensable for a good therapeutic process. Components that are related to the clinical inpatient setting (i.e., setting and organization of the clinic; code of conduct) were also highlighted.

**Conclusions:**

Patients’ and health professionals’ definitions of what constitutes a good treatment entails a wide array of aspects. The clinical setting is seen to offer unique components that are emphasized to have a healing effect.

**Supplementary Information:**

The online version contains supplementary material available at 10.1186/s12913-022-07834-5.

## Introduction

The quality of psychotherapeutic treatments is framed in the context of evidence-based medicine [1]. Consequently, symptom improvement is one of the key criteria to judge a psychotherapy as good. However, recent debates in psychotherapy research question the relevance of symptom improvement as sole indicator of the quality of psychotherapeutic treatment [2–4]. According to this line of argument, additional criteria for improvement need to be considered, including changes in quality of life and functioning, sustainability of achieved changes, cost-effectiveness, availability and acceptability, specificity of used methods/approaches, and the agreement with ethical obligations [2, 3]. Undisputable, a good treatment should be in line with the three essential components of evidence-based practice: acknowledging the relevance of research evidence regarding treatment effectiveness, clinical expertise of the treatment provider, and the patient perspective [5].

While there is a wealth of research providing evidence for psychotherapy’s effectiveness, less research is available that examines treatment providers’ and patients’ views about what contributes to high quality of psychotherapeutic treatment. Qualitative insights have the potential to complement the available evidence from quantitative psychotherapy outcome research, and to allow for a more accurate evaluation of the quality of psychotherapy.

Previously published meta-analyses of qualitative studies [6, 7] reveal that patients experience the following components as most helpful in the process of psychotherapy: (a) awareness/insight/self-understanding; (b) behavioral change/problem solution; (c) empowerment; (d) relief; (e) exploring feelings/ emotional experiencing; (f) feeling understood; (g) patient involvement; (h) reassurance/support/safety; and (i) personal contact. Interestingly, and as already suggested by Elliott [8], most of these categories are linked to the therapeutic alliance. This is empirically supported by a statistically significant association between the patient’s perceived quality of the patient-clinician relationship and the treatment efficacy in face-to-face psychotherapy [9].

Despite the presence of qualitative approaches that examine the impact of helpful events in psychotherapy, the focus of these studies mainly lies on the individual face-to-face therapy setting and is thus conducted among outpatients. However, there exists some evidence that inpatients and outpatients differ in their perspectives about the definition of a good treatment (e.g. [10]), which is also reflected by a survey study that found that inpatients emphasized the inpatient setting and the environment of the clinic – among other factors – to be beneficial during the psychiatric treatment [11]. To understand what constitutes a good treatment in the inpatient setting, the scope of investigation needs to be enlarged. Knowledge about the specific characteristics of what is considered a good treatment in the inpatient setting is important for at least two reasons. First, paying attention to patients’ individual definitions and treatment targets is key since they may substantially impact patients’ satisfaction at the end of psychotherapy [12, 13]. Second, patients and health professionals may have different views on which factors they consider important during the course of care. But, a shared understanding is a significant element of the healing process, which contributes to the feelings of being understood [14, 15].

Therefore, this qualitative study set out to explore patients’ and health professionals’ views regarding the main dimensions of a good treatment in inpatient care.

## Methods

### Study design

Fifteen guideline-based interviews with patients and health professionals were conducted in a Swiss inpatient clinic in 2018. Participants were contacted by the research team and were invited to take part in a qualitative interview, before a standardized e-mail with further details about the project followed. Additionally, standardized occupational and socio-demographic data was collected with a short master data sheet. The interviews took place in the inpatient setting. The average interview duration was 36 min (range = 24 min. to 58 min.).

Participation was voluntary and patients and health professionals were informed that a withdrawal of their participation was possible at any time without negative consequences. Data transcription was completely anonymized. The study procedure and interview guide were reviewed by the Local Ethics Committee, Zurich, Switzerland, and judged as a project which does not fall within the scope of the Human Research Act (HRA).

### Study participants

The medical director of the clinic advertised the study among patients and health professionals. Participants were selected to ensure a naturalistic representation of the clinical sample. In line with the model of deliberate sampling for heterogeneity [16], the goal was to represent a wide range of research objects in the sample. Different subsamples of people involved in the inpatient setting were interviewed: one subsample of patients (*N* = 5), one subsample of psychiatrists (*N* = 5) and one subsample of other health professionals (i.e., nursing staff (*N* = 3), and occupational therapists (*N* = 2)). We did not define additional inclusion or exclusion criteria. Participants were not remunerated for their participation in the study.

### Setting

The qualitative interviews were conducted in a private Swiss inpatient clinic with a special focus on treatment of affective and adjustment disorders. The clinic has 70 beds and is located in a rural setting. The average length of stay at the clinic is between 6 and 8 weeks. The multimodal treatment approach within the clinic focuses on psychotherapy and on pharmacological treatment, the former including individual therapy sessions led by psychiatrists. Patients can attend various therapy programs that are offered by occupational therapists, encompassing mindfulness-based body therapies (e.g., Shiatsu, Feldenkrais, Qi-Gong, Mindfulness-Based Stress Reduction) as well as creative therapies (e.g., Art Therapy, Music Therapy, Occupational Therapy) in individual as well as group settings. Nursing is provided on an individual basis and the interaction with the patients is based on the concept of milieu therapy [17].

### Interview procedure

The interviews were conducted by a trained interviewer (CL) using a semi-structured interview guide. The interview guide was developed by a workgroup on the basis of an extensive literature search. The guide contained four questions on what is experienced as important and helpful during treatment (Table 1). The interviews explored the individuals’ concepts of a good treatment in the clinical setting. The interviews were audio-taped and verbatim transcripts were written. Quotations from the interviews have been translated from Swiss German to English.

**Table 1:** Main topics of interview guide for semi-structured interview

	Patients	Health professionals
**(1) Experience of a good treatment in general**	“When you think about your stay at the clinic – can you tell me how a good treatment is characterized for you?”	“When you think about your patients – can you tell me how a good treatment is characterized for you?”
**(2) Example of a good treatment**	“Can you give me an example or describe a key moment of a good treatment?”	“Can you give me an example or describe a key moment of a good treatment?”
**(3) Ideal case**	“For you personally, how would an ideal treatment look like?”	“What would you wish for yourself and your patient in an ideal treatment?”
**(4) Open question**	“Would you like to add anything we didn’t cover today?”	“Would you like to add anything we didn’t cover today?”

*Note*. Further questions were added in the sense of a semi-structured interview

### Qualitative analysis

**Fig. 1:**
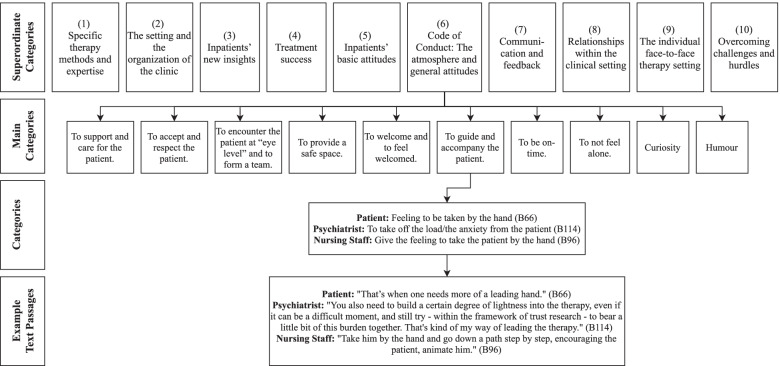
Coding procedure

For data analysis, the online software QCAmap (https://www.qcamap.org) was used. The interviewee’s details from the semi-structured interviews have been evaluated by the content analysis according to Mayring [18]. We chose Mayring’s approach because it is a widely and successfully used method (e.g. [19–22]); and because it is considered a rather economic approach, for example in contrast to grounded theory [22]. Furthermore, we applied an inductive data-driven approach since it allows the examination of core themes for a phenomenon with limited existing theory or research literature [23]. We performed a multistage analytic process ( [18]; see Fig. 1). First, we formulated a research question, focusing on our main topic of what constitutes a good treatment. Second, we defined the level of abstraction a priori, referring to theoretical considerations. Third, we were working through the text material line by line. As soon as a text passage fitted the overall theme, a category was constructed. Fourth, after working through a significant amount of the material (i.e., 15%) we revised the whole category system. This was done by two authors (CL; SaB) and led to some minor revisions. Fifth, we worked through the whole material with the same rules (i.e., category definition and level of abstraction). Sixth, we grouped the final list of categories into main categories. Seventh, we did the inter-coder agreement. For this, the text material was given to a third coder (NH) who had insight into all content-analytical rules. Two authors (SaB; NH) compared the two coding schemes, debated disagreements, and reached consensus on one scheme. Disagreements were resolved with a third author (CL). Finally, we also conducted frequency analyses of the category occurrences in the text material.

## Results

### Sample characteristics

The overall proportion of female interviewees were higher than the proportion of male interviewees. Further characteristics of the interview sample are presented in Table 2.

**Table 2:** Characteristics of interview sample

	Patients (*N* = 5)	Psychiatrist (*N* = 5)	Other health professionals (*N* = 5)
Sex (female:male)	2:3	3:2	4:1
Age (mean; SD)	53.0y; 5.8y	54.4y; 10.3y	51.8y; 1.6y
Interview duration	35 min	34 min	35 min
Diagnosis	2 x F33.1 (Major depressive disorder, recurrent, moderate) 2 x F32.1 (Major depressive disorder, single episode, moderate) 1 x F32.2 (Major depressive disorder, single episode, severe without psychotic features)		
Practice experience (mean; SD)		23y; 8y	18y; 5y

*Note*. *SD* standard deviation; other health professionals are: nursing staff (*N* = 3) and occupational therapists (*N* = 2)

### Qualitative analysis

In the 15 interviews, 546 text passages were identified and summarized in 269 categories. These categories were subsumed in 56 main categories, which were grouped in 10 superordinate categories (see Table 3). An overview of all superordinate categories, main categories and categories is provided in the supplement (eTable 1). eTable 1 also specifies which categories stem from which subsample and shows the number of quotations per category. In the following, we present the 10 superordinate categories. The presented order of the superordinate categories is not linked to the magnitude of quotations within the categories.

1. Specific therapy methods and expertise.

**Table 3:** Superordinate Categories with corresponding Main Categories

Superordinate Categories	Main Categories
1. Specific therapy methods and expertise.	Specific components of the therapy
	Animal assisted therapy
	Physical movement in therapy
	Group therapy settings
	Learning new strategies in therapy
	Expertise and experience of the therapist in his method
2. The setting and the organization of the clinic.	Organization of the clinic
	Possibility to switch the therapist and health professional
	Inpatient setting
	Transfer phase / preparation for everyday life
3. Inpatients’ new insights.	Insights
	Goals
	Development process
	Resources
	Changed behavior
	Implementation of what has been learned in therapy
	Trust in oneself
4. Treatment success.	Treatment success
	Experiences of success
5. Inpatients’ basic attitudes.	Willingness / motivation
	Hope
	Expectations
	Patience
6. Code of Conduct: The atmosphere and general attitudes.	To support and care for the patient
	To accept and respect the patient
	To encounter the patient at “eye level” and to form a team
	To guide and accompany the patient
	To welcome and to feel welcomed
	To provide a safe space
	To be on-time
	To not feel alone
	Humor
	Curiosity
7. Communication and feedback.	Good and transparent communication style between the health professional and the patient
	Good communication style within a group of patients
	Non-verbal communication
	Good communication style in the professional team
	Feedback
8. Relationships within the clinical setting.	The importance of other patients
	Someone is here 24/7
	Practical interactions
	To have a good relationship in the clinical setting
9. Individual face-to-face therapy setting.	Feeling understood in the therapy
	Non-judgmental acceptance in therapy
	To have a balance between emotional closeness and distance in the therapy
	To resonate with the therapist or to have a good match
	To have an (initial) bond in the therapy
	To have a therapeutic relationship that is based on trust
	To have a therapeutic relationship that is constant
	To train how relationships work
	To find a good balance between autonomy and care
	Self-disclosure of the therapist
10. Overcoming challenges and hurdles.	To overcome resistance: Uncomfortable moments are part of every treatment
	To confront and endure difficult moments
	To talk openly about difficulties in the therapeutic relationship
	To learn something as a psychiatrist from difficult situations

Patients valued the importance of specific therapy components and methods such as animal-assisted therapy, occupational therapy, and relaxation-based therapies for the experience of a good treatment.

- Patient: And so, I notice clearly how it helps me to learn to consciously relax the body. What I find very important is that I notice early if I am in a stressful situation (B131).

Most patients also described the acquisition of new strategies in therapy (e.g., tools, coping strategies, daily protocols) as an important process. These strategies were often related to a change in the handling of symptoms. As an example, some of the patients noted that they learned how to cope with problems, how to achieve a certain frustration tolerance, and how to handle their symptoms more effectively.

Some health professionals stated that disorder-specific knowledge is essential for the provision of a disorder-based treatment.

- Psychiatrist: For example, disorder-specific knowledge is required so that I know how to treat anxiety disorders. That I know what is evidence-based, which is very likely to lead to improvement. Or that I know, for example, whether I have to do exposure exercises, leading to a habituation (B116).

What is more: health professionals further emphasized that their own experiences and expertise are crucial for a good treatment. In this context, some interviewees, i.e., also patients, stressed the impact of supervision and therapists’ training.

(2) The setting and the organization of the clinic.

Especially patients and the nursing staff held the opinion that the inpatient setting allows to have a distance from the problem area, enables a daily structure and provides the time to work on the problematic issues.

- Nursing Staff: That they [the patients] have a structure. This can be a professional-therapeutic structure or a structure of their own (B105).

Another topic that was often mentioned by patients, the nursing staff and psychiatrists included the possibility to switch the psychiatrist during the stay at the clinic.

- Patient: There are different people here, with different backgrounds, different interests and that is why it is good that there are different therapists. And you always have the possibility to say: Sorry, that is not true for me, I would like to change [the psychiatrist] (B299).

Furthermore, the organisation of the transfer phase and the preparation for everyday life at the end of the stay is considered crucial for a treatment to be good. This guarantees a sustainable improvement. Some interviewees also made practical recommendations for the transition phase (e.g., making role plays).

(3) Inpatients’ new insights.

Patients and health professionals acknowledged that a good treatment is characterized by a process of self-reflection, development and discoveries that result in the possibility to make new experiences.

- Occupational therapist: So, what I also think is nice, and what I really think is good in a treatment is when the patient understands something about himself and his situation (B46).

New insights have also been linked to changed behaviour, such as the possibility to make new experiences. For example, a patient concluded that “it can be positive to react differently” and that it is a new experience to “consciously not retreat from other people” (B127).

(4) Treatment success.

Psychiatrists outlined that a good treatment is also defined by the reduction of symptoms and the overall treatment success.

- Psychiatrist: Therapeutic success could be, for example, symptom reduction. This is a good therapy for both patients and therapists when the symptoms are decreasing (B56).

Likewise, patients also emphasized that the experience of success is mandatory for a treatment to be good. One participant, for example, mentioned appreciating to “realize that I have taken a step forward and that I have done something” (B9).

(5) Inpatients’ basic attitudes.

All subgroups listed some basic attitudes that the patient must bring into the process in order to increase chances for a good treatment. A core component that was stressed by many interviewees was patients’ willingness and motivation.

- Nursing Staff: A good treatment is guaranteed if the patient can get involved, if s/he is motivated, if s/he can be won and if s/he can also work on herself/himself (B92).

Besides, patients, psychiatrists, and occupational therapists also mentioned that patience is indisputable for a successful therapeutic procedure. Moreover, one occupational therapist also claimed that health professionals themselves have to be patient and that it is crucial to meet patients where they stand:

- Occupational therapist: I think it is important to make room for stagnation, and to not just focus on a methodological goal. It is about responding to the person at this specific moment (B310).

Various interviewees stressed the importance of inpatients’ attitudes that are directed towards the future, i.e., the states of hope and positive expectations. Both aspects were emphasized, i.e., that it is beneficial if the patient is hopeful and optimistic about the future – but also if the health professional provides hope.

(6) Code of Conduct: The atmosphere and general attitudes.

All subsamples agreed that there exist some general attitudes, which are indispensable for a good treatment. The feeling of support and care as well as an accepting and respectful attitude were often mentioned spontaneously at the beginning of the interview:

- Nursing Staff: So, from a patient’s perspective, the most important thing is that I want to be cared for (B260).

The same nurse continued by saying that “the experience to be taken seriously here and to be respected” (B259) is also closely linked to positive changes. Likewise, patients noted that it is an ensuring feeling to “be in good hands” (B368) and that the staff has the ability to make the patient feel comfortable.

In terms of the dynamic between patients and health professionals, interviewees agreed that it is important to work as a team during the therapeutic process and to encounter the patient at “eye level”.

- Patient: And conversely, one can say that these people and the health professionals, the nurses and the physicians, who encounter one at eye level, these are the ones that one really feels are doing good for oneself and that there are good conversations and this also means a successful treatment (B291).

In addition to this partnership-based understanding of therapy, interviewees also highlighted the need to guide and accompany the patient.

- Psychiatrist: I always say, compare psychotherapy to a mountain hike together. That I’m accompanying a patient. I can’t take the backpack, but I’m accompanying, maybe one can say, here is a short-cut, this is a different path, a different perspective (B114).

Whereas psychiatrists and occupational therapists emphasized that a safe space provides the possibility to talk freely and without fear, the nursing staff outlined that a safe space enables patients to (re-)assess themselves and their situation.

- Occupational therapist: So, the setting we work in needs to be safe for the patient. So that s/he is not in danger, has no fear of consequences, that s/he takes me for granted, that s/he knows I’m sticking around (B39).

Presumably, this feeling of secure bonding provides space for humour and curiosity, which are other attitudes that were frequently mentioned. These states were sometimes explicitly perceived as therapeutic tools themselves: A psychiatrist, for example, stated that humour “is an expression of relaxation, of distancing oneself from problems, from heavy feelings, fear or whatever” (B157).

On a more practical level, patients appreciated punctuality. One participant, for example, mentioned how important it was to him that “he [the psychiatrist] was on time “and that “he [the psychiatrist] asked whether we should get started” (B205).

(7) Communication and feedback.

Patients and health professionals agreed: communication and feedback are essential in a good therapeutic process. Patients emphasized the importance of a clear, respectful, as well as open communication style with the health professional. Psychiatrists added that a good communication style can be trained in the therapeutic session. All subsamples brought up the topic and importance of feedback. Whereas health professionals appreciated to receive feedback from patients in order to reflect on their own practice, patients exchange feedback with other patients regarding their own therapy process.

- Occupational therapist: I always ask patients when they say goodbye, i.e., in the last session, if they received what they needed. Or if they achieved what they needed; I find it very exciting, what they say then (B351).

According to the nursing staff and occupational therapists, a good communication style is not only required in the individual face-to-face therapy setting, yet also within a group of patients and the professional team.

- Nursing Staff: Well, if it’s something that concerns many people or the whole group, then for me this is something that I address in the patients’ morning round (B272).

In this quote, we see that beyond verbal communication, non-verbal and ‘atmospheric’ components are also seen as important in the exchange.

- Psychiatrist: This is exactly the moment of the first encounter, eye contact, body posture, mimic. And sometimes the nonverbal message of the patient is, yes, I’m looking forward to seeing you again (B228).

(8) Relationships within the clinical setting.

Aspects of the clinical setting that contribute to a supportive relationship were mostly stressed by patients and the nursing staff. The vast majority underlined the importance of the presence of fellow patients. Patients greatly acknowledged the feeling of belongingness. They also specified that they appreciate to talk about everyday things as well as more specific conversations about one’s mood or progress with fellow patients.

- Patient: Then, what I have experienced very positively here is of course the community of the patients. It has been really wonderful here and that has helped a lot (B207).

Also strongly related to the clinical setting was the common statement that it is a relief that someone is always here when needed.

- Nursing Staff: And as a patient I want to have the possibility that someone is present 24 h a day (B281).

Likewise, the nursing staff emphasized the advantages of the practical interactions that occurred during the stay at the clinic such as doing the dishes together, drinking a coffee, or going for a walk.

- Nursing Staff: For example, at the barbecue evening, where at the end you stand together in the kitchen doing the dishes and washing up. That’s something that somehow connects (B324).

(9) The individual face-to-face therapy setting.

Common themes of the face-to-face therapy setting encompassed basic therapeutic attitudes such as the feeling of being understood, a non-judgemental acceptance, and trust.

- Psychiatrist: But perhaps also a certain expression of the emotional understanding of the patient, of his concern, of his problem, so empathy belongs to the good treatment, feeling oneself in, putting oneself in, understanding, not only cognitively (B223).

Likewise, many patients valued that they were able to express themselves in a manner that is true to who they are, and that they were accepted in this way. Notably, while some psychiatrists emphasized that one should not be afraid of intimacy in the therapy, others stressed that an adequate balance between emotional closeness and distance is key in therapy.

- Occupational therapist: And I have to be separated from patients to a certain degree, but I also have to be tangible for the patient, for his world (B347).

Furthermore, health professionals outlined that they have the duty to guide and lead patients.

- Occupational therapist: With a few patients it is quite clear that they are very needy or need structure – I am certainly more supportive and suggest something that would give them some structure. And for a critical patient, who is able to structure himself well, yes, then I can ask him/her what would be of interest now (B394).

Finally, some interviewees mentioned that it is beneficial if health professionals provide insights about their own daily life, without being afraid of self-disclosure.

- Patient: That he [the therapist] also often brings an example from his life. (…) He then tells me how he reacts and that it was difficult for him as well in the beginning, but that it is possible to implement it differently than I do at the moment. I then realize that this is very ‘human’ – and this feels good (B217).

(10) Overcoming challenges and hurdles.

Even though positive feelings are appreciated by patients and health professionals, a good treatment is also characterized by the possibility to overcome challenges and hurdles together.

- Psychiatrist: As soon as something is activated, these are also important moments. That is, where it might be unpleasant for the patient, maybe for me, those are probably also the most important moments for the therapeutic process (B75).

Interviewees also specifically proposed how to handle difficult moments: they should be accepted, rather than avoided. Psychiatrists also suggested that one should openly talk about difficulties and ruptures in the therapeutic relationship.

- Psychiatrist: Yes, she [the patient] just had some kind of crisis on Monday after talking to me. And then we talked about it on Tuesday and yes, somehow this crisis was also helpful. It wasn’t my intention, but it did cause something (B237).

Interestingly, psychiatrists said that they also learn from difficult situations during the therapeutic relationship – and that they therefore have to face their own insecurities from time to time.

- Psychiatrist: Sometimes I gain self-awareness and sometimes it also affects the relationship, which can be difficult. This is also important, not always pleasant but still good. Yes, that I can then also expand my potential for improvement – that is good therapy (B353).

In conclusion, our findings indicate that a good treatment entails much more than symptom improvement as measured by efficacy and effectiveness outcomes according to patients and health professionals. Likewise, our interviews revealed that a good treatment in the inpatient setting is best seen as multidimensional, including patient-specific (i.e., inpatients’ new insights; inpatients’ basic attitudes), treatment-specific (i.e., the specific therapy methods and expertise; treatment success; individual face-to-fact therapy setting), relationship-based (i.e., relationships within the clinical setting; communication and feedback; overcoming challenges and hurdles), and setting-specific (i.e., the setting and the organization of the clinic; code of conduct: the atmosphere and general attitudes) aspects. Notably, many core aspects are probably generalizable to other treatment settings (e.g., outpatient treatment, home-based care-giving), whereas other aspects are directly related to the inpatient setting (e.g., the supportive contact with fellow patients, practical interactions and teamwork).

## Discussion

In this qualitative study we aimed to explore patients’ and health professionals’ views on the main dimensions of what constitutes a good treatment in the inpatient setting. Some categories were linked with the processes that each individual patient undergoes during therapy, others were related to specific therapy components. We also found that a good therapeutic alliance is one of the core aspects in order for a treatment to be considered good. Finally, interviewees of our study also mentioned that the clinical inpatient setting itself has a positive impact on the treatment outcome.

Previous research confirms the relevance of feelings of support and care as early as in the first psychotherapy session [24]. Participants in our study valued health professionals with non-judgmental and accepting attitudes. Psychiatrists and occupational therapists also highlighted that a supportive therapeutic relationship should be based on trust and continuity. These core attitudes of acceptance, respect, trust, support and care, as well as the feeling of being understood have been described as determinants of the quality of the therapeutic relationship (e.g. [24, 25]). Meta-analyses confirm the positive association between diverse aspects of the patient-clinician relationship and health outcomes [9, 26, 27].

Furthermore, patients and health professionals stressed the importance of shared decision making and of forming a team. This is in line with the partnership-based model in psychotherapy [28]. Complementary to this, interviewees emphasized the need to guide and accompany the patients during their personal process and development. It has been proposed that clinician’s responsiveness to the needs of their patients enables patients to be guided to move through points that are painful and challenging [15]. Patients value a safe space where they have the possibility to talk freely and without fear. Notably, other qualitative studies in the field of mental health services also found that patients highlighted the impact of confidentiality (e.g. [29, 30]). To sum up, interviewees in our study stressed two different layers that complement and enrich each other: first, to be on the same page and to encounter each other at “eye level”; second, to accompany each patient in his/her individual level of neediness, and to provide a confidential and safe space.

Another related dimension that was pointed out by the different subgroups is the process of feedback. Health professionals stated that they encourage patients to give them feedback so that their own practice can be refined and adjusted. In psychotherapy research, it is undisputable that feedback loops substantially enhance the treatment gains, and systems have been proposed to routinely integrate feedback loops into mental health care [31]. Interestingly, inpatients in our study utilized fellow patients for an internal feedback process – by comparing themselves with other patients. This result reflects the well-known phenomena of social comparison in groups [32], and is in line with the social comparison perspective which claims that heterogeneous groups of patients have the potential to positively influence the efficacy of therapeutic programs [33].

Our study also revealed that a good treatment does not have to be perceived good all the time. On the contrary: the interviewees highlighted that a supportive therapeutic relationship provides the fundament to overcome challenges and hurdles. Psychiatrists emphasized that they themselves learn from difficult situations. Researchers acknowledge that there are no definitive indicators of when an emotional process during psychotherapy is adaptive or maladaptive – mainly because the emotional response largely depends on contextual factors [34]. In this sense, our findings underline the notion that addressing positive along with negative emotions may contribute to the success of psychotherapy.

### Unique aspects of what is considered a good treatment within inpatient clinical settings

While patients and health professionals widely agreed on core attitudes that are indispensable for a good treatment in face-to-face therapeutic setting, some of the mentioned aspects are to be considered unique and specific for the inpatient setting. Notably, many of these aspects are under-examined and under-represented in the currently available research literature.

The inpatient setting, by its very nature, enables to engage in numerous human interactions. Most strikingly, interviewees underlined the impact of the presence of fellow patients. First, patients evoke the feeling of belongingness to each other. People who have had similar experiences can relate and are able to provide more authentic empathy and understanding [35]. The process of providing and receiving support has been proposed to empower and increase self-esteem, self-efficacy and self-management [36]. Second, fellow patients provide a confidential space where one can talk about personal issues but also about topics that are unrelated to the clinical condition. Finally, patients and the nursing staff emphasized the role of companionship and friendship. In this line of reasoning, peer support has been suggested to support the positive aspects of human interaction [37], leading to beneficial moments during the inpatient psychiatric treatment [11]. In the very few qualitative studies that also examined naturally occurring peer support, additional topics such as sharing material goods [38] and “stepping in” (e.g., providing protection against aggressive behavior of others) were mentioned.

Furthermore, patients and the nursing staff emphasized the value of practical interactions. Patients also mentioned that it can be relieving to talk about everyday things. A review on the nurse-patient interaction in acute inpatient mental health units reported a similar finding: The nursing staff has the ability to utilize everyday situations for a meaningful interaction with patients; “nothing is too small for the psychiatric nurse to respond to” [39].

Finally, the nursing staff as well as occupational therapists emphasized that a good communication style within the professional team is necessary for a fruitful relationship and, in turn, the beneficial interaction with the patients. This also includes a regular interdisciplinary exchange with other health professions. The need to work as a team has also been acknowledged by other qualitative examinations [25].

### Limitations

This study has several limitations. First, one shortcoming of qualitative studies is that the external validity of the results is often limited. In particular, our study took place at a Swiss private clinic that is applying the so-called contextual model of psychotherapy [4]. Therefore, the generalizability of the findings to other cultural and clinical settings is questionable. Also, findings from the inpatient setting are not transferrable to the outpatient setting. Second, with our sample size of 15, only a small number of people are sampled from the different subgroups. However, it was not our intention to specifically represent each subgroup, but rather to have a general impression from themes that are specific for the  inpatient treatment process. Third, there was a restricted selection of participants in the study as only inpatients with affective and adjustment disorders were treated in the clinic. It might be that different findings would have been found in participants with other diagnoses. Fourth, the findings of our study do not reveal whether the features that the respondents appreciated as important for a good treatment actually contribute to good treatment outcomes. Finally, the choice to apply a content analysis according to Mayring has the limitation that only thematic descriptions are produced which stands in contrast to other approaches which enable to generate explanatory theories (e.g., grounded theory) [40, 41]. Nevertheless, we believe that for the aim of our study (i.e., to explore participants’ views regarding a good treatment in the inpatient setting), a content analysis was most suitable.

## Conclusion and future directions

This study indicates that the question of what constitutes a good treatment goes far beyond the concept that a treatment has to be efficacious and effective in order to be considered good and that the exclusive focus on symptom-related health outcomes needs to be reconsidered.

According to our interviewees, a good treatment in the inpatient setting is best seen as multidimensional, including patient-specific, treatment-specific, relationship-based, and setting-specific aspects. These components seem to interact with each other, forming a unique structure that is greater than the sum of the parts. The inpatient setting itself encompasses much more than the direct interaction between two people – fellow patients, practical interactions and teamwork are part of the everyday life. Our results show that the bond with other patients has a positive impact on the feeling of belongingness and that the beneficial effect of teamwork outside of the actual therapeutic sessions should not be underestimated. Although our findings are in line with other qualitative approaches, these constituents of a good treatment are widely under-represented in efficacy studies and theoretical approaches. We claim that naturally occurring peer support as well as teamwork should be recognized components of a good treatment in the inpatient stay. It is known that peer support often prolongs over the inpatient stay and is assumed to have maintaining positive effects. Further research on what is considered a good treatment in the clinical setting needs to be conducted with larger samples in different locations in order to validate the current findings, also in the long-term.

## Supplementary Information

**Additional file 1: eFigure 1.** Inductive development of categories according to Mayring. **eTable 1.** Superordinate categories, main categories and categories.

## Data Availability

The dataset used during the current study are available from the corresponding author on reasonable request.

## Abbreviation

HRA: Human Research Act
