# Supplementary material for: Greater than the sum of the parts: a qualitative content analysis of what constitutes a good treatment in the inpatient setting
Source: BMC Health Serv Res. 2022 Apr 27;22:565. doi: 10.1186/s12913-022-07834-5 (PMC9044573; doi:10.1186/s12913-022-07834-5)
Supplement: Supplementary file 1 — Additional file 1: eFigure 1. Inductive development of categories according to Mayring. eTable 1. Superordinate categories, main categories and categories. [file 12913_2022_7834_MOESM1_ESM.docx]

**SUPPLEMENTARY MATERIALS**

**Table of Contents**

**eFigure 1**. Inductive development of categories according to Mayring

**eTable 1.** Superordinate categories, main categories and categories

**eFigure 1. Inductive development of categories according to Mayring**

**eTable 1. Superordinate categories, main categories and categories**

| **Superordinate Categories** | **Main Categories** | **Categories** | **Amount of Quotations** |
| --- | --- | --- | --- |
| 1. Specific therapy methods and expertise. | Specific components of the therapy | P:B4 animal assisted therapy: tours with the therapy dog | 1 |
|  |  | P:B7 occupational therapy | 2 |
|  |  | P:B125 relaxation therapies: in order to come to rest | 1 |
|  |  | P:B131 body therapy: to recognize signs from your body e.g. when you get into a stressful situation | 4 |
|  |  | P: B188 shiatsu | 2 |
|  |  | P:B190 mindfulness-based therapies | 1 |
|  |  | P:B191 mindfulness-based therapies: in order to come to rest or to be aware of all senses (hearing, smelling etc.) | 3 |
|  |  | P:B221 body therapy: learning to relax the body | 3 |
|  |  | P:B303 body therapies | 1 |
|  |  | PS:B88 exposition therapy: to overcome a fear and make good experiences | 1 |
|  | Animal assisted therapy | P:B5 animal assisted therapy: feeling that the animal gives something back to you | 2 |
|  |  | P:B202 animal assisted therapy: a direct, concrete contact to the animal | 1 |
|  | Physical movement in therapy | P:B11 animal assisted therapy: movement because of the dog | 1 |
|  | Group therapy settings | P:B204: discussion in groups | 1 |
|  | Learning new strategies in therapy | P:B33 mediation of tools how to get better | 9 |
|  |  | P:B67 do and learn things in the therapy to get energy back | 4 |
|  |  | P:B193 to learn how to cope with problems | 1 |
|  |  | P:B302 to achieve a certain frustration tolerance in the clinic and to learn how to cope with it | 1 |
|  |  | PS:B57 better handling of the symptoms (by the patient) | 3 |
|  |  | PS:B89 daily protocol about positive experiences; self-verbalisation | 1 |
|  |  | PS:B91 mediation of tools how to get better | 6 |
|  |  | NS:B95 mediation of tools how to get better | 3 |
|  |  | OT:B65 mediation of tools how to get better | 1 |
|  | Expertise and experience of the therapist in his method | P:B387 therapists training | 0 |
|  |  | PS:B116 knowledge/scientific underpinning of the intervention/methods | 1 |
|  |  | OT:B49 ability to form a good relationship through experiences and expertise | 2 |
|  |  | OT:B50 a good sense through experiences and the personality of the therapist | 1 |
|  |  | OT:B51 the application of new knowledge and experiences through therapist training | 1 |
|  |  | OT:B342 therapists experience to know when he can challenge the patient | 0 |
|  |  | OT:B343 therapists experience to know what patient needs | 0 |
|  |  | OT:B346 patient can rely on therapists’ experience | 0 |
| 2. The setting and the organization of the clinic. | Organization of the clinic | P:B206 good time schedule for the activities | 1 |
|  |  | P:B253 regular breaks for the staff, so that they can guarantee high quality | 1 |
|  | Possibility to switch the therapist and health professional | P:B219 possibility to switch their health professional (nurse) | 1 |
|  |  | P:B299 possibility to switch the therapist during stay at the clinic | 1 |
|  |  | PS:B85 possibility to switch the therapist during stay at the clinic | 8 |
|  |  | NS:B287 to also be able as a patient to give up a relationship; switch the health professional/therapist | 1 |
|  | Inpatient setting | P:B1 distance from the problem area | 9 |
|  |  | P:B2 daily structure | 9 |
|  |  | P:B68 having the time to recover | 2 |
|  |  | P:B200 flexible pressure on the daily structure | 2 |
|  |  | P:B213 having the time to work on problems | 2 |
|  |  | NS:B105 having structure | 1 |
|  |  | NS:B261to make the patient feel that he has time to recover | 1 |
|  |  | NS:B328 to have an insight view into the habits and daily practices of the patient | 1 |
|  | Transfer phase / preparation for everyday life | P:B245 a good preparation process for leaving the clinic (transfer) to achieve a sustainable improvement (e.g. through practical inputs) | 4 |
|  |  | P:B300 the practical support of the therapist to implement the future ideas of life plans | 1 |
|  |  | P:B390 meet patients to plan activities for the time after clinic stay | 0 |
|  |  | PS:B336 be able to cope with problems at the end of therapy | 3 |
|  |  | NS:B290 ability to show the patients the alternatives that exist in their lifes after treatment | 2 |
|  |  | NS:B327 to make role plays with the patient e.g. of the problematic situation | 1 |
|  |  | OT:B352 end of therapy: if patient is able to try out new things | 0 |
| 3. Inpatients’ new insights. | Insights | P:B170 when the patient understands something in the course of the therapy process/makes discoveries | 3 |
|  |  | P:B243 the understanding of the maladaptive mechanisms of one's own behaviour in order to change them sustainably | 2 |
|  |  | PS:B74 when the patient understands something in the course of the therapy process/makes discoveries | 7 |
|  |  | PS:B78 when the therapist makes discoveries about himself | 2 |
|  |  | PS:B178 when the therapist discovers resources of the patient | 1 |
|  |  | PS:B235 having a magic moment in therapy | 2 |
|  |  | OT:B46 when the patient understands something in the course of the therapy process/makes discoveries | 4 |
|  |  | OT:B311 if the patient him/herself is able to identify needs | 4 |
|  |  | OT:B312 making discovering’s by means of experiences (mostly nonverbal) | 6 |
|  |  | OT:B313 self-reflection/talking about own discoveries with the (occupational-)therapist | 3 |
|  | Goals | P:B37 occupational therapy: goal-focus | 1 |
|  |  | PS:B90 the formulation of what one expects from a therapy | 1 |
|  |  | PS:B120 goal-orientation of a therapy: to assess where you stand regarding your goals | 4 |
|  |  | PS:B154 setting goals for the future | 1 |
|  |  | PS:B337 therapy goal: recovery but not healing | 0 |
|  |  | NS:B97 goal-orientation of a therapy: setting goals and working on them | 2 |
|  |  | NS:B330 when patients are able to achieve their own goals | 1 |
|  | Development process | P:B194 if you reach a new orientation through self-reflection | 5 |
|  |  | P:B196 when the patient does his/her own developments/discoveries | 1 |
|  |  | P:B197 occupational therapy: changing the focus from the outcome to the process ("joy of doing" a task) | 1 |
|  |  | PS:B79 possibility to improve your own potential as a therapist | 1 |
|  |  | PS:B142 when the patient does his/her own developments/discoveries and the therapist feels superfluous | 3 |
|  |  | PS:B169 when the patient does his/her own developments/discoveries | 6 |
|  |  | NS:B94 when the patient does his/her own developments/discoveries | 2 |
|  |  | OT:B328 when the patient has an understanding for himself again | 1 |
|  |  | OT:B319 when the patient has a more open view (does not focus on his suffering) | 1 |
|  |  | OT:B320 when the patient is more communicative | 1 |
|  | Resources | PS:B77 when resources of the patient are discovered | 5 |
|  | Changed behavior | P:B127 the possibility to make new experiences and to see (positive) consequences | 1 |
|  |  | P:B218 therapy context as a space to try things out | 1 |
|  |  | PS:B198 if the patient changes his usual patterns | 1 |
|  | Implementation of what has been learned in therapy | P:B195 implementation what he/she (patient) has learned in the therapy into real life | 6 |
|  |  | PS:B232 implementation what he/she (patient) has learned in the therapy into real life | 2 |
|  |  | NS:B98 implementation what he/she (patient) has learned in the therapy into real life | 1 |
|  | Trust in oneself | PS:B144 improvement in self-confidence (patient) | 2 |
|  |  | PS:B165 when the patient is confident after a therapy | 3 |
|  |  | NS:B109 improvement in self-confidence (patient) | 1 |
|  |  | OT:B55 feeling of strength; e. g. to go home and take challenges | 1 |
|  |  | OT:B317 when the patient regains his self-confidence | 2 |
| 4. Treatment success. | Treatment success | P:B121 variety of interventions that actually help | 2 |
|  |  | PS:B56 treatment success: symptom reduction | 3 |
|  |  | PS:B73 if the intervention succeeds; e. g. the cognitive restructuring | 2 |
|  |  | PS:B80 long-term success / change | 4 |
|  | Experiences of success | P:B9 occupational therapy: if a significant progress has been achieved | 2 |
|  |  | PS:B71 if the patient makes progress | 3 |
| 5. Inpatients’ basic attitudes. | Willingness / motivation | P:B13 if the patient is motivated | 1 |
|  |  | P:B173 if the patient takes an active part in the treatment | 7 |
|  |  | P:B388 staff encourage patient to become active | 0 |
|  |  | PS:B59 if the patient takes an active part in the treatment | 4 |
|  |  | PS:B174 if the patient is motivated | 2 |
|  |  | NS:B92 if the patient is motivated | 1 |
|  |  | NS:B93 if the patient takes an active part in the treatment | 3 |
|  |  | NS:B276 the patient's own willingness to make changes/to get treated | 2 |
|  |  | NS:B283 to be able to fully engage with the treatment (patient) | 1 |
|  | Hope | PS:B143 the therapist providing hope for the patients future | 2 |
|  |  | PS:B166 if the patient is optimistic and hopeful about the future | 1 |
|  |  | PS:B236 symptom reduction contributing to hope | 1 |
|  | Expectations | PS:B141 providing a realistic prognosis for the patient | 1 |
|  |  | PS:B150 realistic expectations for the patient | 1 |
|  | Patience | P:B124 occupational therapy: endurance on a task | 4 |
|  |  | PS:B153 to have the patience in a therapy | 1 |
|  |  | OT:B310 endure difficult moments | 1 |

| 6. Code of Conduct. | To support and care for the patient | P:B22 support of nursing staff; e.g. be able to get rid of something that puts a strain on you | 4 |
| --- | --- | --- | --- |
|  |  | P:B180 to feel supported | 4 |
|  |  | P:B368 to feel in good hands | 0 |
|  |  | P:B292 the ability of the staff (therapist, nurses etc) to make the patient feel comfortable | 1 |
|  |  | PS:B133 to feel well cared for (patient) | 3 |
|  |  | PS:B62 to feel supported (patient) | 3 |
|  |  | PS:B359 to let the patient feel that she/he is in good hands | 0 |
|  |  | NS:B260 to make the patient feel well cared for | 5 |
|  |  | NS:B275 to care for the patient (even if it is none of my business) | 1 |
|  |  | NS:B280 to give the patient what he / she wants at the moment | 1 |
|  |  | NS:B288 looking for alternatives how the patient can be helped if something does not work | 1 |
|  | To accept and respect the patient | PS:B132 acceptance of the symptoms (by the patient) | 2 |
|  |  | PS:B149 flexibility on the part of the therapist | 1 |
|  |  | PS:B356 therapists’ ability to acknowledge patients views | 0 |
|  |  | PS:B81 openness / different perspectives or opinions about the success of a therapy | 1 |
|  |  | NS:B259 to convey to patients the feeling that they are being taken seriously and respected | 4 |
|  |  | OT:B340 therapist respects the otherness | 0 |
|  |  | OT:B45 openness / unbiasedness on the part of the therapist | 2 |
|  | To encounter the patient at “eye level” and to form a team | P:B291 encounter the patient at "eye level" | 3 |
|  |  | PS:B112 give the patient the feeling that they (patient and therapist) form a team where the patient is accompanied by the therapist | 2 |
|  |  | OT:B308 therapy at the same "eye level" | 2 |
|  | To guide and accompany the patient | P:B66 feeling to be taken by the hand | 1 |
|  |  | PS:B114 to take off the load/the anxiety from the patient | 2 |
|  |  | NS:B96 give the feeling to take the patient by the hand | 1 |
|  | To welcome and to feel welcomed | P:B179 to feel welcomed | 1 |
|  |  | P:B370 clinic staff having warm manners | 0 |
|  |  | PS:B111 a good introductory process from the therapist as a person | 2 |
|  | To provide a safe space | P:B376 therapist is able to stabilize patient | 0 |
|  |  | P:B377 if therapist provides space for patients needs | 0 |
|  |  | PS:B146 providing safety in therapy | 2 |
|  |  | PS:B147 providing containment to the patient | 1 |
|  |  | PS:B110 protected space to talk about problems | 7 |
|  |  | NS:B279 to provide safety/orientation for the patient | 1 |
|  |  | NS:B380 caring and respectful environment that helps the patient to open up | 0 |
|  |  | OT:B39 to feel safe in the course of the therapy (occupational-therapist) | 2 |
|  |  | OT:B306 having the space for a good therapeutic relationship | 1 |
|  |  | OT:B314 to provide a space for the patient to find his needs or what he wants or does not want | 3 |
|  |  | OT:B345 patient dares to come out of his shell | 0 |
|  | To be on-time | P:B205 therapist is on time | 1 |
|  | To not feel alone | PS:B355 patients feeling of not being alone | 0 |
|  | Humor | P:B240 humorous conversation style between patient and therapist | 1 |
|  |  | B389 other patients: humorous interaction | 0 |
|  |  | PS:B157 humor/laughing in the therapy | 1 |
|  |  | NS:B333 humor | 1 |
|  |  | OT:B338 humor/laughing in the therapy | 0 |
|  | Curiosity | PS:B227 if patient and therapist look forward to the next session | 1 |
|  |  | PS:B229 patient being curious about continuation of therapy | 1 |
|  |  | PS:B357 therapist having good encounters with interesting people | 0 |
| 7. Communica-tion and feedback. | Good and transparent communication style between the health professional and the patient | P:B239 honest conversation style between therapist and patient | 1 |
|  |  | P:B241 profound talks between therapist and patient | 1 |
|  |  | P:B247 very clear but also respectful communication between therapist and patient | 2 |
|  |  | P:B248 a good communication between patient and health professional | 1 |
|  |  | P:B293 be transparent with the patient (what is happening in the therapy) | 2 |
|  |  | P:B378 if therapist is able to take former topics up | 0 |
|  |  | P:B181 be able to talk to a specialist | 1 |
|  |  | P:B379 open communication of therapist | 0 |
|  |  | PS:B139 the use of understandable language for the patient | 1 |
|  |  | PS:B230 patients training of communication in therapy | 1 |
|  |  | PS:B362 the use of a common language | 0 |
|  |  | NS:B108 good communication between patient and therapists/health professionals | 1 |
|  |  | NS:B326 valuable conversations with the patients about their problems | 2 |
|  |  | NS:B274 to be informed about the patients and to let the patients feel that | 1 |
|  |  | NS:B383 be transparent in treatment | 0 |
|  | Good communication style within a group of patients | P:B397 very clear but also respectful communication between therapist and patient | 0 |
|  |  | NS:B272 open communication with patients | 1 |
|  | Non-verbal communication | P:B249 a good nonverbal communication between patient and health professional | 1 |
|  |  | P:B189 shiatsu: touches that do you good | 1 |
|  |  | PS:B152 use and understanding of non-verbal communication | 1 |
|  |  | PS:B224 non-verbal, affirmative reactions of understanding from the therapist | 1 |
|  |  | PS:B228 non-verbal communication towards the therapist expressing positive feelings | 1 |
|  | Good communication style in the professional team | NS:B270 open communication within the team | 1 |
|  |  | NS:B273 exchange with the team about a (patient's) situation before facing the situation itself | 2 |
|  |  | OT:B305 good communication in a professional team | 2 |
|  | Feedback | P:B128 other patient: estimation of how much one did get better through comparison with others | 4 |
|  |  | PS:B233 patients training of giving and receiving feedback | 1 |
|  |  | PS:B334 possibility to get qualitative feedback from the patient | 0 |
|  |  | PS:B87 possibility to get feedback from patients to reflect oneself as a therapist or the therapy itself | 9 |
|  |  | NS:B107 get a feedback from the patient | 2 |
|  |  | NS:B271 to be able to give honest feedback to each other in the team | 1 |
|  |  | OT:B351 if (occupational-)therapist asks patient for feedback | 0 |
|  |  | OT:B396 other patients good experience help patient to open up | 0 |
| 8. Relationships within the clinical setting. | The importance of other patients | P:B129 other patient: to provide support to other patients | 1 |
|  |  | P:B207 other patients: belongingness | 4 |
|  |  | P:B208 other patients: confidential space to talk about problems | 2 |
|  |  | P:B209 other patient: activities together | 1 |
|  |  | P:B210 possibility to see what others have created | 1 |
|  |  | P:B214 other patients: talk about unproblematic things | 1 |
|  |  | P:B216 to realize that other patients have the same problems but can cope with them | 1 |
|  |  | P:B220 other patient: possibility to choose the company | 1 |
|  |  | P:B23 patients training in therapy of having relationships | 4 |
|  |  | P:B25 other patient: feeling of not being alone with problems | 5 |
|  |  | P:B296 other patients: mindfulness towards each other | 1 |
|  |  | P:B369 other patients: help to acclimatize to the new environment | 0 |
|  |  | P:B373 meditation: feeling of calmness within the group | 0 |
|  |  | P:B374 other patients: interesting to hear other patients stories | 0 |
|  |  | NS:B257 patient feels accepted and valued by other patients | 1 |
|  |  | NS:B268 to observe and support the patient in the introduction process with other patients | 1 |
|  |  | NS:B269 other patients support especially during the introduction phase e. g. in order to be easier integrated | 1 |
|  |  | NS:B381 other patients: belongingness | 0 |
|  | Someone is here 24/7 | NS:B281 to give the patients the feeling that someone is here 24/7 | 3 |
|  | Practical interactions | NS:B324 practical interactions with the patient (such as doing the dishes or drinking a coffee together) | 4 |
|  | To have a good relationship in the clinical setting | P:B126 good relationship between patient and therapist | 3 |
|  |  | PS:B61 good relationship between patient and therapist | 9 |
|  |  | NS:B103 good relationship between patient and therapist | 1 |
|  |  | OT:B42 good relationship between patient and therapist | 1 |
| 9. Individual face-to-face therapy setting. | Feeling understood in the therapy | P:B34 feeling understood by the therapist | 3 |
|  |  | PS:B223 empathical understanding of the patient | 1 |
|  |  | PS:B161 if the therapist is able to recognize and meet the patient's needs in stages | 2 |
|  |  | PS:B162 if the therapist is able to grasp insecurities/anxieties of the patient and helps reduce them | 1 |
|  |  | PS:B226 therapists understanding of patients difficulties to end the therapy | 1 |
|  |  | PS:B138 developing a common understanding of the patient's disorder | 2 |
|  |  | NS: B384 to reflect on negative feelings towards patients | 0 |
|  |  | OT:B64 feeling understood by the therapist | 1 |
|  |  | OT:B53 the ability to let the patient know that he is heard | 2 |
|  | Non-judgmental acceptance in therapy | P:B122 the understanding from the therapist without a judgement | 1 |
|  |  | P:B250 possibility to behave the way you feel in front of the clinic staff | 2 |
|  |  | P:B251 unevaluating, non-resentful attitude of the care staff towards the patient | 1 |
|  |  | PS:B177 the understanding of the therapist without a judgement | 1 |
|  | To have a balance between emotional closeness and distance in the therapy | PS:B148 not to be afraid of intimacy in the therapy (therapist) | 1 |
|  |  | OT:B347 therapist is able to simultaneously be aware of patient and himself | 0 |
|  |  | OT:B348 therapists adequate balance between distance and closeness | 0 |
|  | To resonate with the therapist or to have a good match | P:B298 good match between therapist and patient | 2 |
|  |  | P:B386 personality of the therapist | 0 |
|  |  | P:B391 therapist is able to adapt intellectually to patient | 0 |
|  |  | PS:B354 resonate in therapy | 0 |
|  |  | PS:B83 same values between therapist and patient | 1 |
|  |  | PS:B222 therapist mirrors patients body language | 1 |
|  |  | PS:B360 if the therapist is genuinely human | 0 |
|  |  | OT: B339 resonate in therapy | 0 |
|  | To have an (initial) bond in the therapy | P:B215 to feel initially in good hands in the therapy | 2 |
|  |  | PS:B225 initial bond between patient and therapist | 1 |
|  | To have a therapeutic relationship that is based on trust | PS:B358 to form a trustful relationship | 0 |
|  |  | PS:B366 trustful, secure relationship as base to confront difficulties | 0 |
|  |  | OT:B395 trustful, secure relationship as base to confront difficulties | 0 |
|  | To have a therapeutic relationship that is constant | PS:B119 a constant / sustainable relationship offered by the therapist | 4 |
|  |  | PS:B163 after the completion of psychotherapy: being able to let go / send off the patient | 1 |
|  | To train how relationships work | PS:B231 patients training in therapy of having relationships | 1 |
|  |  | PS:B364 to have new positive relationship experiences in therapy | 0 |
|  |  | NS:B286 to work on the relationship between patient and therapist/health professional | 1 |
|  | To find a good balance between autonomy and care | PS:B367 therapists decides what patient needs (not what patient wants) | 0 |
|  |  | NS:B382 a good balance between autonomy and care | 0 |
|  |  | OT:B394 a good balance between being directive and give patient autonomy | 0 |
|  | Self-disclosure of the therapist | P:B217 to get information from the therapist that he knows the problem from his own experience | 1 |
|  |  | OT:B349 disclosing when therapist knows patients’ problem from his own life | 0 |
|  |  | OT:B350 if (occupational-)therapist is able to disclose feelings and moods | 0 |
| 10. Overcoming challenges and hurdles. | To overcome resistance: Uncomfortable moments are part of every treatment | P:B294 uncomfortable moments/overcome a resistance | 2 |
|  |  | PS:B75 uncomfortable moments/overcome a resistance | 13 |
|  |  | OT:B40 uncomfortable moments/overcome a resistance | 6 |
|  | To confront and endure difficult moments | P:B246 ability of the (occupational-) therapist to endure unpleasant/hard situations with the patient | 1 |
|  |  | PS:B86 to confront/overcome difficulties in the relationship between therapist and patient | 4 |
|  |  | OT:B171 if difficult moments are not avoided but rather accepted by the therapist | 1 |
|  | To talk openly about difficulties in the therapeutic relationship | PS:B237 talking about rupture in the therapeutic relationship | 2 |
|  |  | PS:B238 courage of the therapist to speak about difficult topics | 1 |
|  | To learn something as a psychiatrist from difficult situations | PS:B353 if therapists learn something about himself through difficult moments in the relationship | 0 |
|  |  | PS:B344 therapist faces own insecurities | 0 |

*Note*. OT = occupational therapists; PS = psychiatrist; NS = nursing staff; P = patient.
